# Plant soil interactions alter carbon cycling in an upland grassland soil

**DOI:** 10.3389/fmicb.2013.00253

**Published:** 2013-09-10

**Authors:** Bruce C. Thomson, Nick J. Ostle, Niall P. McNamara, Simon Oakley, Andrew S. Whiteley, Mark J. Bailey, Robert I. Griffiths

**Affiliations:** ^1^Centre for Ecology and HydrologyWallingford, Oxfordshire, UK; ^2^Centre for Ecology and HydrologyLancaster, Lancashire, UK; ^3^School of Earth and Environment, The University of Western AustraliaCrawley, WA, Australia

**Keywords:** upland acidic grassland, bacteria, substrate-specific respiration, priming effects, substrate carbon use efficiency, T-RFLP, RNA stable isotope probing, soil organic carbon

## Abstract

Soil carbon (C) storage is dependent upon the complex dynamics of fresh and native organic matter cycling, which are regulated by plant and soil-microbial activities. A fundamental challenge exists to link microbial biodiversity with plant-soil C cycling processes to elucidate the underlying mechanisms regulating soil carbon. To address this, we contrasted vegetated grassland soils with bare soils, which had been plant-free for 3 years, using stable isotope (^13^C) labeled substrate assays and molecular analyses of bacterial communities. Vegetated soils had higher C and N contents, biomass, and substrate-specific respiration rates. Conversely, following substrate addition unlabeled, native soil C cycling was accelerated in bare soil and retarded in vegetated soil; indicative of differential priming effects. Functional differences were reflected in bacterial biodiversity with *Alphaproteobacteria* and *Acidobacteria* dominating vegetated and bare soils, respectively. Significant isotopic enrichment of soil RNA was found after substrate addition and rates varied according to substrate type. However, assimilation was independent of plant presence which, in contrast to large differences in ^13^CO_2_ respiration rates, indicated greater substrate C use efficiency in bare, *Acidobacteria*-dominated soils. Stable isotope probing (SIP) revealed most community members had utilized substrates with little evidence for competitive outgrowth of sub-populations. Our findings support theories on how plant-mediated soil resource availability affects the turnover of different pools of soil carbon, and we further identify a potential role of soil microbial biodiversity. Specifically we conclude that emerging theories on the life histories of dominant soil taxa can be invoked to explain changes in soil carbon cycling linked to resource availability, and that there is a strong case for considering microbial biodiversity in future studies investigating the turnover of different pools of soil carbon.

## Introduction

In terrestrial ecosystems the amount of organic C stored in soil is largely dependent upon a dynamic balance between inputs, mostly from plants (Hopkins and Gregorich, 2005), and respired outputs from soil (Kuzyakov, 2002). Plant communities directly affect soil C storage as they provide a range of C resources, mainly in the forms of root exudates and detritus, which are decomposed at different rates by the soil biota (De Deyn et al., 2008; Paterson et al., 2009). Soil microbial communities are known to be incredibly diverse and are essential for decomposition (Schimel and Schaeffer, 2012), yet there is little evidence that belowground microbial biodiversity affects soil organic matter turnover (Nannipieri et al., 2003). Indeed, it has been suggested that abiotic factors, and not microbial community structure, regulate mineralization rates (Kemmitt et al., 2008). With major advances in understanding global soil microbial biodiversity (Fierer et al., 2009), it is also imperative we improve our understanding of the diversity and activity of soil microbes within a functional context, particularly to elucidate the role of soil biodiversity in the terrestrial C balance and wider sustainability of soils (Bardgett et al., 2008; Paterson et al., 2009).

When studying soil organic matter dynamics, isotope labeled substrate additions often reveal changes in the decomposition rates of unlabeled native soil organic carbon (SOC); a phenomenon termed *the priming effect* (Bingeman et al., 1953). Priming effects can be either positive or negative and are thought to be important in determining a soil's C storage potential (Kuzyakov et al., 2000). Positive priming describes increased native soil organic matter cycling following fresh substrate inputs, whereas negative priming is the converse decline in native cycling following inputs. The underlying mechanisms affecting the magnitude and direction of priming are not yet fully understood, despite many lab and field based studies examining potential factors, such as: vegetation presence (Brant et al., 2006; Guenet et al., 2010); resource availability (Kuzyakov and Demin, 1998; Fontaine et al., 2004a; Kuzyakov and Bol, 2006); and substrate quality and quantity (De Nobili et al., 2001; Pascault et al., 2013). There are further complications as to the source of the native carbon being assessed in priming effects studies, be it from turnover of non-living soil organic matter (termed real priming effects), or from the endogenous metabolism of microorganisms (apparent priming effects) (Dalenberg and Jager, 1981; Bell et al., 2003; Blagodatskaya and Kuzyakov, 2008). We do not enter this debate here, and will henceforth use the term *SOC* to refer to carbon present in both living and non-living components of existing soil organic matter, prior to fresh organic carbon (FOC) addition.

Several recent theories have proposed that a better understanding of the role of soil microbial communities may be required to fully understand priming effects (Fontaine et al., 2003; Fontaine and Barot, 2005; Kuzyakov and Bol, 2006). Fontaine et al. (2003), in particular, detailed hypothetical models based on microbial population dynamics and soil nutrient conditions which placed microbial diversity and activity as a key driver of priming effects. Here, microbial populations with different life histories are considered to respond differently to added carbon inputs, where copiotrophs (*r*-strategists) rapidly utilize the added FOC, whereas oligotroph (*K*-strategist) populations more efficiently channel the energy gained from FOC into degrading SOC. This theory also emphasizes that plants may have an important role in driving soil priming effects as they determine soil fertility, and affect microbial community structure and functional activity by providing FOC inputs of differing resource value (Griffiths et al., 2003; Johnson et al., 2003; Bardgett, 2005).

In Fontaine's theory (Fontaine et al., 2003) it was posited that, after utilizing FOC, particular microbial populations increase in size resulting in a greater turnover of SOC. Therefore, it is supposed that monitoring differences in soil microbial community structure, alongside soil C fluxes, could provide insights into the microbial populations contributing to the direction and magnitude of soil priming effects. Whilst some studies have simply monitored microbial communities in conjunction with soil respiration measures (Falchini et al., 2003; Landi et al., 2006; De Graaff et al., 2010; Guenet et al., 2010), others have traced ^13^C-labeled substrates into lipid biomarkers (Nottingham et al., 2009). Additionally, stable isotope probing (SIP) of nucleic acids has been undertaken to permit more detailed molecular analyses of the diversity of active microbes (Bernard et al., 2007, 2012; Pascault et al., 2013). These few studies have shown that population shifts in active microbes can be associated with changes in soil C turnover, yet more studies are still needed to synthesize and strengthen current theories on the relationships between microbial biodiversity and the cycling of different pools of SOC.

In a previous study we showed that field-based removal of vegetation from an acidic grassland decreased soil resource availability, soil respiration rates and changed bacterial community structure to favor presumed oligotrophic taxa (Thomson et al., 2010). Using these same treatments, here we seek to investigate in more detail the role of vegetation (presence or absence), resource availability and bacterial biodiversity on the specific cycling of labile FOC and native SOC. Three ^13^C labeled substrates will be used to examine how the type of substrate, as a proxy for labile FOC resources, affects the observed patterns. Total bacterial community responses will be assessed and we also seek to explore the use of an RNA-based SIP approach to investigate active communities degrading different C sources, and to further our understanding of soil priming effects.

## Materials and methods

### Field site, soil sampling, and properties

Soil (10 cm depth) was collected in autumn at an upland, grassland experiment located at the Rigg Foot field site, Sourhope, Scotland, UK (GR NT854 196 at 300 m above sea level). In a previous experiment, soil bacterial community structure and respiration rates were found to vary at the field scale as a result of the topography of the experimental site, which led to waterlogging in certain areas (Thomson et al., 2010). Therefore, in this experiment we chose to examine replicates from within a single experimental block. The experimental block comprised of a 10 m^2^ area of control grassland (hereon referred to as vegetated treatment), within which a 4 m^2^ defoliated plot had been established and covered with a permeable black membrane, for 3 years preceding this experiment, to prevent plant growth (hereon referred to as bare treatment). Three replicate monoliths (60 cm × 60 cm) were sampled from the vegetated and bare areas within close proximity of each other to minimize variation in environmental conditions; ensuring that the main difference between treatments was the presence or absence of plants. Samples were transported immediately to the laboratory where they were fresh sieved to 2 mm, roots removed by hand then stored at 4°C until required for analyses and ^13^C substrate addition experiments.

Experimental microcosms were set-up using 100 ml air-tight containers (Lock & Lock, Armorica, Petersfield, UK) modified to include a rubber septum (SubaSeal, Sigma-Aldrich, Poole, UK) for headspace gas sampling. Triplicate microcosms (20 g sieved soil) were established to enable destructive sampling at 0 (prior to substrate addition), 24, 72, and 193 h to examine extracted RNA, and total and functional bacterial communities in vegetated and bare treatments (equaling a total of 78 microcosms). These replicates were also used for CO_2_ sampling on ten occasions (0, 12, 24, 48, 72, 96, 120, 144, 168, and 193 h).

C and N contents were analyzed with an Elementar Vario EL elemental analyser (Elementar Analysensysteme GmbH, Hanau, Germany). Deionized water was added to soil to provide a 1:1 mixture and pH was measured using an HI 8424 pH meter (Hanna Instruments Srl, Italy). Moisture (%) was calculated by drying overnight at 105°C then re-weighing to measure water loss. Microbial biomass measurements were based on analysis of phospholipid fatty acids (PLFAs), using a previously described method (Bardgett et al., 1996). Phospholipids were extracted from 1.5 g soil (fresh weight) and extracts analyzed using an Agilent 6890 Gas Chromatograph (Zebron ZB-5 Capillary GC Column 60 m × 0.32 mm × 0.25 μm). Individual PLFA peaks were identified based on retention times of known bacterial fatty acid standards (Sigma-Aldrich, Dorset, UK). Concentrations of individual fatty acids were calculated using a standard 19:0 peak as a reference. Total soil microbial PLFA concentrations were calculated from all measured PLFAs (15:0, 15:0i, 15:0a, 14:0, 16:0i, 16:0, 16:1, 16:1ω5, 16:1ω7, 17:1ω8, 7Me-17:0, br17:0, 17:0i, 17:0a, br18:0, 18:1ω5, 18:1ω7, 18:0, 19:1, 7,8cy-19:0).

### ^13^C substrate incubation experiment

Fully labeled (99 atom%) ^13^C-glucose, ^13^C-glycine, and ^13^C-phenol (Cambridge Isotope Laboratories Inc. Andover, UK) were weighed and dissolved in distilled H_2_O. The ^13^C labeled substrate solutions were sterilized through a 0.2 μm filter (Sartorius, Göttingen, Germany) and added at a rate of 0.4 mg ^13^C g^−1^ dry soil. For the glucose, glycine and phenol incubations 200, 250, and 220 μl of substrate solution was added, respectively to each microcosm. Filter sterilized, distilled H_2_O was also used as a control treatment. After the addition of substrate solution or distilled H_2_O, soil was stirred once to mix substrates before the initial gas sampling (0 h). Moisture contents were maintained throughout by weighing and rewetting. At each sampling, microcosms were sealed shut and headspace gas samples taken immediately and after 3 h to determine the soil respiratory CO_2_ flux. Microcosms were maintained at approximately 15°C (mean summer temperature) in a temperature controlled room.

### CO_2_ and ^13^C–CO_2_ analyses

CO_2_ measurements were made with a Perkin Elmer Autosystem XL Gas Chromatograph (Perkin Elmer, Waltham, MA, USA), fitted with a flame ionization detector, operated at 350°C. CO_2_ was isothermally separated with nitrogen as the carrier gas flowing at 30 cm^3^ min^−1^ on a 2 m column packed with Poropak Q. The detector response was calibrated using a certified gas standard containing 500 μl l^−1^ CO_2_ in nitrogen (Air Products, Leeds, UK). Between sampling events lids were left open to allow microcosms to vent.

To analyze respired ^13^CO_2_, headspace gas sampled from microcosms was injected into a Trace Gas pre-concentrator unit (Micromass, Manchester, UK), and ^13^C content was subsequently quantified using gas chromatography isotope ratio mass spectrometry (GC-IRMS) (Micromass, Manchester, UK). Analysis was performed at the NERC Life Sciences Mass Spectrometer Facility, CEH Lancaster using their standard protocols (uncertainty better than 0.3%). Abundances of ^13^C were expressed as ^13^C atom% i.e.:

$${}^{13}\text{C atom}\%\;=\;[(R_{\text{sample}})÷(R_{\text{sample}}+1)]\;\times \;100$$

where *R*_sample_ is the ^13^C:^12^C ratio of analyte CO_2_.

### Respiration calculations

The amount of substrate ^13^C respired as ^13^CO_2_–C (mg g^−1^ dry soil h^−1^) was calculated for each sampling event using the equation:

$${}^{13}\text{C from substrate}=\left[\left({\text{respCO}}_{2}\right)÷100\right]\;\times {\;}^{13}\text{​C atom}\%\text{ excess}$$

where respCO_2_ = the flux of total CO_2_–C respired from soil over 3 h at each time point, and ^13^C atom% excess = the ^13^C atom% difference between two measurements taken 3 h apart at each sampling time point.

Rates of unlabeled SOC turnover following substrate addition were calculated using an equation previously described by Fontaine et al. (2004a,b):

$$\text{unlabeled SOC turnover}=\left({\text{tot-substrate}}^{13}\text{C-tot}\_\text{soilresp}\right)$$

where tot-substrate^13^C = total respiration minus ^13^C respired following substrate amendment (mg g^−1^ dry soil h^−1^), and tot_soilresp = total soil respiration from the water control treatment (mg g^−1^ dry soil h^−1^).

### Total bacterial community structure

Nucleic acids were extracted from 0.5 g soil using a previously described method (Griffiths et al., 2000) and were finally re-suspended in molecular-grade H_2_O. Terminal restriction fragment length polymorphism (T-RFLP) analysis of soil bacterial communities was performed using diluted extracted nucleic acids with 16S rRNA gene primers 63F (Marchesi et al., 1998) (fluorescently-labeled with D4 blue dye) (Sigma-Proligo, Dorset, UK) and 519R (Lane, 1991) (MWG Biotech, London, UK). Amplicons were then digested with *Msp*I restriction enzyme (New England Biolabs Inc., Ipswich, MA, USA) prior to fragment analysis using a Beckman Coulter CEQ 2000XL capillary sequencer (Beckman Coulter Corporation, California, USA). Terminal restriction fragment (T-RF) relative abundances were calculated as the ratio between the fluorescence of individual T-RFs and the total integrated fluorescence of all T-RFs.

### Stable isotope analyses of soil RNA

Soil RNA was purified from total nucleic acids extractions using a RNA/DNA minikit (Qiagen, Crawley, UK) according to the manufacturer's instructions. To assess the amount of substrate ^13^C incorporation into soil RNA, ^13^C:^12^C isotope analysis was performed using a method previously described (Manefield et al., 2002a). RNA (1 μg) was cut with sucrose to provide a minimum C content of 25 μg. Samples were then freeze-dried for 16 h prior to combustion to CO_2_ using a Carlo-Erba N1500 Elemental Analyser (Carlo-Erba, Valencia, CA, USA). Abundances of ^13^C were expressed as δ^13^C values, using the following equation:

$$\delta^{13}\text{C }\left(\%\right)\;=\;[(R_{\text{sample}}÷R_{\text{standard}})-1]\;\times \;1000$$

where *R*_sample_ and *R*_standard_ are the ^13^C:^12^C ratio of soil extracted RNA and Pee Dee Belemnite standard, respectively.

Substrate ^13^C incorporated into RNA (μg μg^−1^ RNA) was calculated for each sampling event using the following equation:

$${}^{13}\text{C substrate in RNA}=[\left({\text{RNA}}^{13}\text{C atom}\%÷\text{100}\right)\;\times \;25]-{\text{sucrose}}^{13}\text{C}$$

where RNA ^13^C atom% = ^13^C atom% of RNA; sucrose^13^C = the amount of ^13^C (μg) in sucrose standard used to cut extracted RNA; and 25 = minimum C content in μg.

To investigate microbial substrate C use efficiency, we examined the amount of substrate ^13^C assimilated into RNA compared to the amount of substrate ^13^C respired, based on a previously described calculation (Frey et al., 2001; Brant et al., 2006):

$$\text{substrate C use efficiency}=[\left({\text{RNA}}^{13}\text{C}\right)÷({\text{RNA}}^{13}\text{C}+\sum {\text{substrate}}^{13}\text{C})]$$

where RNA^13^C = the amount of ^13^C incorporated into extracted RNA (μg μ g^−1^ RNA), and substrate^13^C = cumulative substrate-specific respiration (μg g^−1^ dry soil).

### RNA stable isotope probing

SIP and denaturing gradient gel electrophoresis (DGGE) analysis were performed similarly to Manefield et al. (2002a). Extracted RNA was subjected to isopycnic density gradient centrifugation in caesium trifluoroacetate gradients containing deionised formamide. Gradients were loaded with 500 ng of extracted RNA and spun at 136,000 × g for 42 h at 20°C. Following centrifugation, samples were fractionated for 30 s per fraction at a flow rate of 3.3 μl s^−1^, resulting in a total of 20 fractions per sample. RNA was precipitated from each gradient fraction by incubating at −20°C with isopropanol followed by centrifugation at 16,000 × g for 30 min at 4°C. Finally, fractions were dried under vacuum and RNA dissolved in RNase-free water.

Precipitated RNA from density gradient fractions was reverse transcribed using reverse primer 519r (MWG Biotech, London, UK) and avian myeloblastosis virus reverse transcriptase (Promega, Southampton, UK). cDNA was then amplified using bacterial 16S rRNA gene primers GC338F and 519r (MWG Biotech, London, UK). DGGE analysis was performed with a 10% (wt/vol) acrylamide gel containing a denaturant gradient of 30–60%. Denaturing gradient gels were cast and run using the Ingeny PhorU2 system (Goes, The Netherlands) at 60°C and 200 V for 8 h. Approximate amounts of RT-PCR product were loaded into each lane on the gel to examine active bacterial communities. Gels were subsequently stained with SYBR gold nucleic acid gel stain (Molecular Probes, Invitrogen, Paisley, UK), then visualized by UV trans-illumination.

### Statistical analyses

Cumulative respiration data, RNA ^13^C incorporation and substrate C use efficiency were examined for significant differences between treatments with a One-Way analysis of variance (ANOVA) combined with Tukey's *post hoc* testing with a family error rate set at 5, using MINITAB release 14 (MINITAB Inc.). Statistical analyses of soil bacterial communities were performed with the vegan library (Oksanen et al., 2009) of the R software package (R Core Development Team, 2005). Briefly, a Bray-Curtis distance matrix of between sample dissimilarities was calculated and subsequently represented through two-dimensional non-metric multidimensional scaling (NMDS), using the metaMDS function. Differences in communities were quantified by permutational multivariate analysis of variance (PERMANOVA) using the adonis function, and group dispersions (beta diversity) were further assessed using the betadisper function. To assess differences in the relative abundances of particular terminal restriction fragments (T-RFs) between treatments, similarities of percentages (SIMPER) analysis (Clarke, 1993) was performed using the PAST statistical package (http://folk.uio.no/ohammer/past).

## Results

### Effects of vegetation removal on soil properties and biomass

After 3 years without plant cover, soil C and N contents, and microbial biomass were significantly lower in the bare soil treatment compared to the vegetated soil (*P* < 0.05, *F* = 70.49, *F* = 63.78, and *F* = 23.27 for soil C and N contents, and microbial biomass, respectively). The C:N ratio was less in vegetated soil, although not significantly so. Additionally, there were no significant differences in % moisture and soil pH between the two soil treatments (Table 1).

**Table 1:** **Mean soil properties in vegetated and bare soils**.

	**C (g g^−1^ soil)**	**N (g g^−1^ soil)**	**C:N**	**Soil pH**	**Moisture (%)**	**Microbial biomass (PLFA μg g^−1^ dry soil)**
Vegetated	0.119 (0.001)	0.0094 (0.0002)	12.70 (0.33)	4.81 (0.03)	54.00 (0.58)	102.40 (9.11)
Bare	0.097 (0.002)	0.0070 (0.0002)	13.57 (0.26)	4.77 (0.01)	53.67 (1.67)	57.47 (1.91)
*P*	0.001	0.001	0.109	0.22	0.86	0.008
*F*	70.49	63.78	4.24	0.17	0.04	23.27

*Standard error of the mean (SEM) in parentheses (n = 3). Significant differences calculated using One-Way ANOVA*.

### Gross respiration is higher in vegetated soil

**Figure 1:**
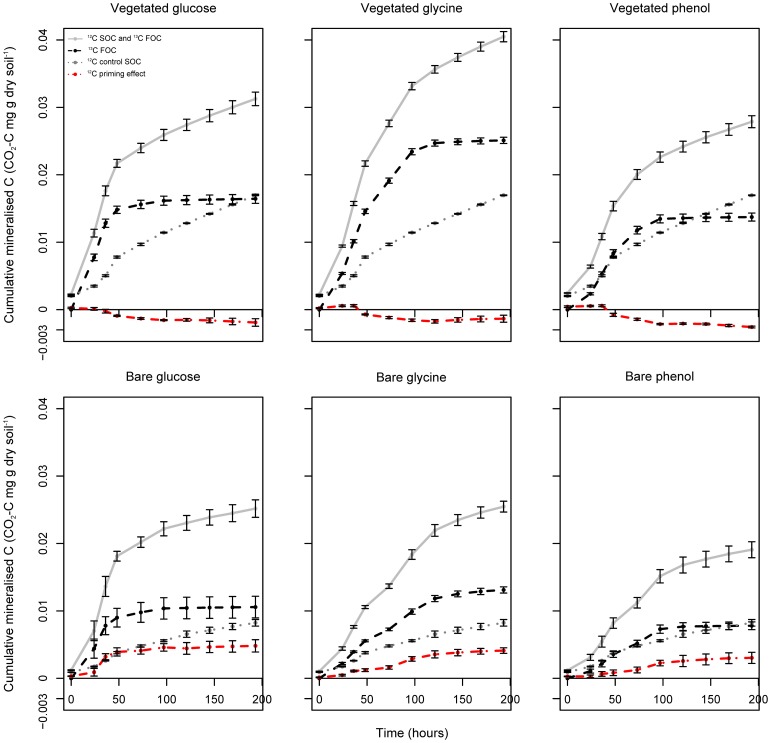
**Labeled FOC and unlabeled SOC turnover in vegetated and bare soils amended with labile ^13^C substrates.** Mean total respiration (^12^C SOC and ^13^C FOC), substrate-specific respiration (^13^C FOC) and ^12^C priming effects in vegetated and bare soils following the addition of ^13^C-labeled glucose, glycine, and phenol. Priming effects were based on unlabeled SOC turnover rates after FOC addition, relative to ^12^C SOC mineralization in the control soil. Error bars are SEM (*n* = 3).

Cumulative soil basal respiration was significantly higher (*P* < 0.05, *F* = 315.47) in vegetated soil compared to the bare (Figure 1), by the end of the experiment. Similarly, total respiration following substrate addition was greatest in vegetated soil regardless of which substrate was added (*P* < 0.05, glucose *F* = 13.74; glycine *F* = 184.84; phenol *F* = 35.17). By examining the shape of the respiration curves, rates were shown to differ depending on substrate added. Following glucose addition, respiration was greatest at the start of the experiment, tailing off toward the end. Yet, for glycine and phenol lower levels of respiration were sustained for a longer period of time, and displayed a biphasic respiration response (data not shown). In both treatments, mean cumulative total respiration rates were ranked in the following order: glycine > glucose > phenol. In vegetated soil, mean cumulative total respiration was significantly higher following glycine addition compared to the glucose and phenol incubations; however, there was no significant difference between glucose and phenol mean cumulative total respiration (based on confidence interval ranges following a One-Way ANOVA with Tukey's *post hoc* test). Contrastingly, in bare soil, phenol total respiration was significantly less than glucose or glycine, and there were no significant differences between the glucose and glycine treatments. Basal respiration was more than two times greater in the vegetated control treatment than the bare, though this magnitude of difference was not observed in total respiration rates. This emphasizes that C processing, in terms of total amounts of respiration following substrate addition, differed to that of native organic matter respiration between the two treatments.

### Increased FOC mineralisation in vegetated soil

In order to specifically examine proportions of ^13^C labeled FOC and unlabeled SOC mineralized, headspace gases were analyzed using GC-IRMS to determine the specific amount of respired ^13^CO_2_. By the end of the incubation, cumulative substrate-specific respiration was significantly higher in vegetated soil compared to bare soil for all substrate additions (*P* < 0.05, ^13^C-glucose *F* = 11.32; ^13^C-glycine *F* = 336.72; ^13^C-phenol *F* = 51.14) (Figure 1). Over the entire duration of the experiment, ^13^C-substrates were mineralized in the following order: glycine > glucose > phenol for both vegetated and bare soils. In vegetated soil, total cumulative ^13^C-substrate mineralization was significantly different between all substrates (based on confidence interval ranges following a One-Way ANOVA with Tukey's *post hoc* test). In bare soil, however, there were found to be no significant differences between glucose and glycine, and glucose and phenol mineralization rates. Both substrate-induced and substrate-specific respiration data revealed very similar patterns in terms of treatment differences between bare and vegetated soils, and soil respiration responses to substrate amendment.

### Accelerated SOC turnover in bare soils following FOC addition

The difference in ^12^C respiration between substrate amended soils and the water controls was inferred to have arisen from the decomposition of SOC in response to the addition of FOC (the priming effect). The direction and intensity of unlabeled SOC turnover varied with soil treatment, FOC type and incubation time (Figure 1, red lines). For all FOC additions there was a significant difference in mean cumulative SOC turnover between vegetated and bare soils (*P* < 0.05, glucose *F* = 39.29; glycine *F* = 68.03; phenol *F* = 44.58). For vegetated soil, irrespective of the FOC used, addition of labile ^13^C led to a cumulative decrease in SOC mineralization compared to the control incubation (11, 7, and 15% mean decrease for glucose, glycine and phenol, respectively). Conversely, in the bare soils, addition of low molecular weight FOC brought about a cumulative increase in SOC decomposition relative to the water control (61, 51, and 38% mean increase for glucose, glycine and phenol, respectively). Within each soil treatment, the cumulative amount of SOC mineralized over the duration of the experiment was unaffected by the type of FOC added (vegetated soil *P* = 0.21; bare soil *P* = 0.29). This shows that regardless of the nature of FOC added, the presence or absence of vegetation had significant consequences for unlabeled SOC turnover; with consistent positive priming (accelerated unlabeled SOC turnover) in bare soils and negative priming (retarded unlabeled SOC turnover) in vegetated soils.

### Total bacterial community structure

**Figure 2:**
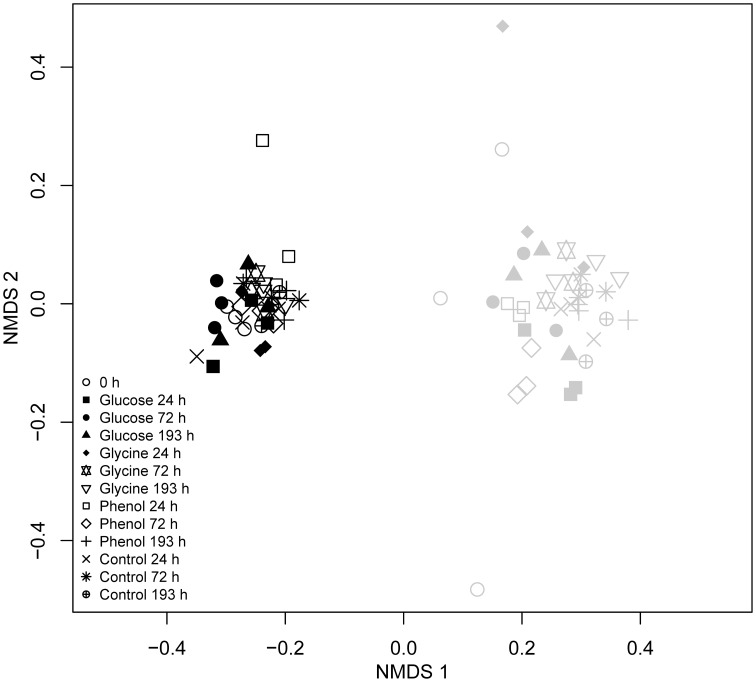
**Effects of substrate addition upon dominant soil bacterial populations in vegetated and bare soils.** Two-dimensional NMDS ordination analysis of bacterial T-RFLP data from soils incubated with ^13^C-labeled glucose, glycine, and phenol, and the water control treatments at 0, 24, 72, and 193 h. Differences in the relative abundances of *Alphaproteobacteria* and *Acidobacteria* T-RFs were found to account for the majority of differences between vegetated and bare soil communities. Bacterial communities from vegetated and bare soils are denoted by black and gray symbols, respectively.

Two-dimensional NMDS analysis was performed to explore any differences in bacterial community structure between vegetated and bare soils across the incubations (Figure 2). The presence or absence of plants was shown to be the main factor responsible for community differences as vegetated and bare soils were clearly separated along the first axis. The effect of the vegetated and bare treatments on soil bacterial community structure was also shown to be significant when analyzed with PERMANOVA (*R*^2^ = 0.53, *P* < 0.05). NMDS analysis also showed little change in bacterial communities after exposure to FOC or at different sampling events throughout the incubation. However, in the bare treatment there were distinct differences in the bacterial communities analyzed prior to FOC addition as these samples grouped separately from all other samples. Additionally, bare soil communities across all substrate additions and time points were significantly more variable than vegetated soil communities (betadisper, *P* < 0.05).

**Figure 3:**
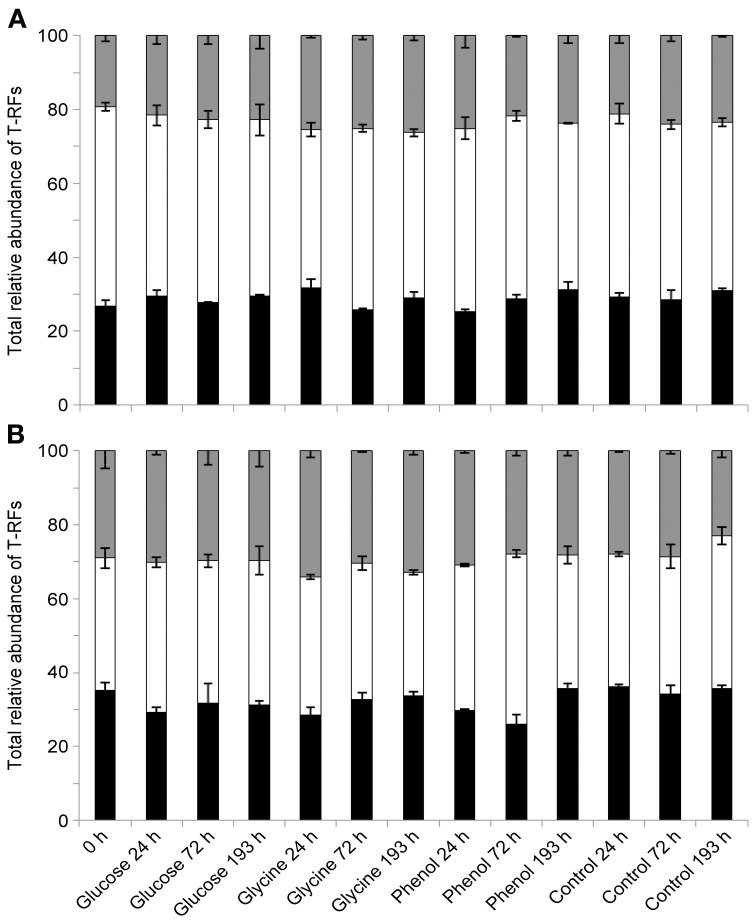
**Relative abundances of dominant bacterial taxa over time, following labile substrate additions.** Totaled relative abundances of *Acidobacteria* T-RFs (black), *Alphaproteobacteria* T-RFs (white), and T-RFs representing all other taxa (gray) in vegetated **(A)** and bare **(B)** soils (*n* = 3, error bars are SEM).

SIMPER analysis highlighted that the T-RFs which accounted for approximately 55% of the dissimilarity between the vegetated and bare soil bacterial communities were 53, 55, 76, 77, 78, 97, 99, 110, 111, 112, 113, 227, 229, and 396 nucleotides (n.t.) in length. Using data from a previous *in silico* endonuclease restriction digest of 16S rRNA gene clone libraries from vegetated and bare soils (Thomson et al., 2010), we could confidently identify all but two (97 and 99 n.t.) of these T-RFs at the class level (Table 2). Only minor variations in the relative abundances of these taxa occurred over time and between substrate additions in vegetated and bare soils, with the main treatment differences being a significantly greater relative abundance of *Alphaproteobacteria* T-RFs in vegetated soil (*P* < 0.05, *F* = 104.18) and *Acidobacteria* T-RFs in bare soil (*P* < 0.05, *F* = 16.86) (Figure 3). Furthermore, the mean ratio of *Alphaproteobacteria* to *Acidobacteria* T-RFs was significantly higher (*P* < 0.05, *F* = 51.64) in vegetated soil than bare soil, with values of 1.69 and 1.22, respectively.

**Table 2:** **Analysis of dominant bacterial taxa in vegetated and bare soils**.

	**Contribution**	**Cumulative %**	**Vegetated soil abundance**	**Bare soil abundance**	**ID**
55	3.185	8.767	0.0798	**0.143**	*Acidobacteria*
113	2.645	16.05	**0.133**	0.08	*Alphaproteobacteria*
112	2.098	21.82	0.00455	**0.0457**	*Alphaproteobacteria*
227	1.799	26.77	**0.0632**	0.0275	*Acidobacteria*
99	1.682	31.41	0.00867	**0.0422**	Unclassified
53	1.343	35.1	0.0595	**0.0828**	*Acidobacteria*
78	1.089	38.1	**0.0334**	0.0123	*Alphaproteobacteria*
76	1.071	41.05	**0.0311**	0.0104	*Alphaproteobacteria*
110	0.9677	43.71	**0.11**	0.0986	*Alphaproteobacteria*
111	0.9456	46.32	0.00647	**0.0163**	*Alphaproteobacteria*
97	0.9057	48.81	0.0033	**0.0213**	Unclassified
229	0.8751	51.22	**0.0477**	0.0314	*Acidobacteria*
77	0.8027	53.43	**0.0189**	0.00328	*Alphaproteobacteria*
396	0.6228	55.14	**0.0332**	0.0258	*Alphaproteobacteria*

*Mean relative abundances of dominant T-RFs contributing to approximately 55% of the total dissimilarity (n = 39). Results from SIMPER analysis are listed in order of decreasing importance. Numbers highlighted in bold indicate whether a particular T-RF was more abundant in the vegetated or bare soil. The identities of T-RFs are based on 16S rRNA gene clone libraries from a previous study (Thomson et al., 2010)*.

### ^13^C incorporation into soil RNA

**Figure 4:**
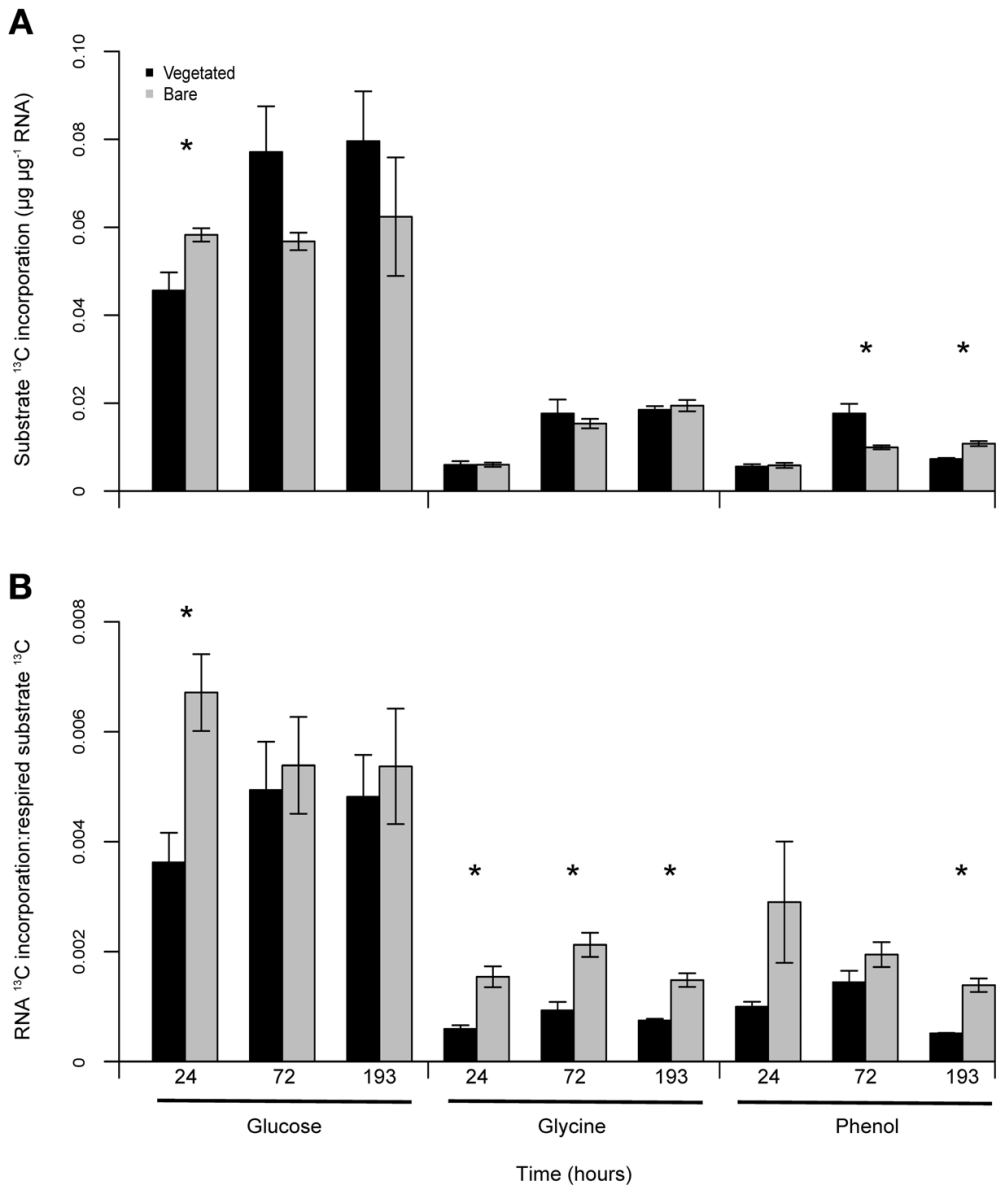
**Differences in substrate ^13^C utilisation in vegetated and bare soils.** Mean assimilation of glucose, glycine and phenol ^13^C into RNA extracted from soil (μg^13^C μg^−1^ RNA) **(A)**. Glucose ^13^C incorporation was greater than the other FOC additions, although there were no consistent effects between vegetated and bare soils. Mean FOC use efficiency, displayed as the ratio of RNA ^13^C incorporation to respired substrate (RNA ^13^C:^13^CO_2_-C μg g^−1^ dry soil) **(B)**. FOC was consistently utilised more efficiently in bare soil. Error bars are SEM (*n* = 3). Significant differences denoted by ^*^.

Dynamic measurements of ^13^C incorporation into extracted soil RNA were performed to assess microbial utilization of added FOC. Differences in rates of ^13^C incorporation were particularly pronounced for glucose amended soils, as the total ^13^C incorporation into RNA from glucose was three- to four-fold greater than glycine or phenol ^13^C. Throughout the experiment there was no consistent difference in RNA ^13^C incorporation rates between vegetated and bare soils; significant differences were only observed 24 h after glucose addition (*P* < 0.05, *F* = 8.31), and 72 h and 193 h after phenol addition (*P* < 0.05, *F* = 11.77; *P* = 0.004, *F* = 31.92) (Figure 4A).

To further investigate FOC dynamics across treatments, we calculated and contrasted substrate ^13^C use efficiencies, determined as the ratio of RNA incorporated ^13^C to respired substrate ^13^C (Figure 4B). Here we assume RNA incorporation rates reflect the amount of FOC being assimilated into cellular components as opposed to being respired for catabolic metabolism (Manzoni et al., 2012). In terms of the differences between applied substrates, glucose ^13^C was consistently utilized more efficiently than the other two substrates independently of vegetation presence or absence. Additionally, mean utilization efficiencies were consistently higher in bare soil independent of FOC type, with significant differences 24 h after glucose addition (*P* < 0.05, *F* = 12.25); 24, 72, and 193 h following glycine addition (*P* < 0.01, *F* = 22.28; *P* = 0.01, *F* = 19.81; *P* < 0.01, *F* = 33.01); and 193 h after phenol addition (*P* < 0.01, *F* = 51.12).

### Sip reveals no difference between “total” and “active” communities

**Figure 5:**
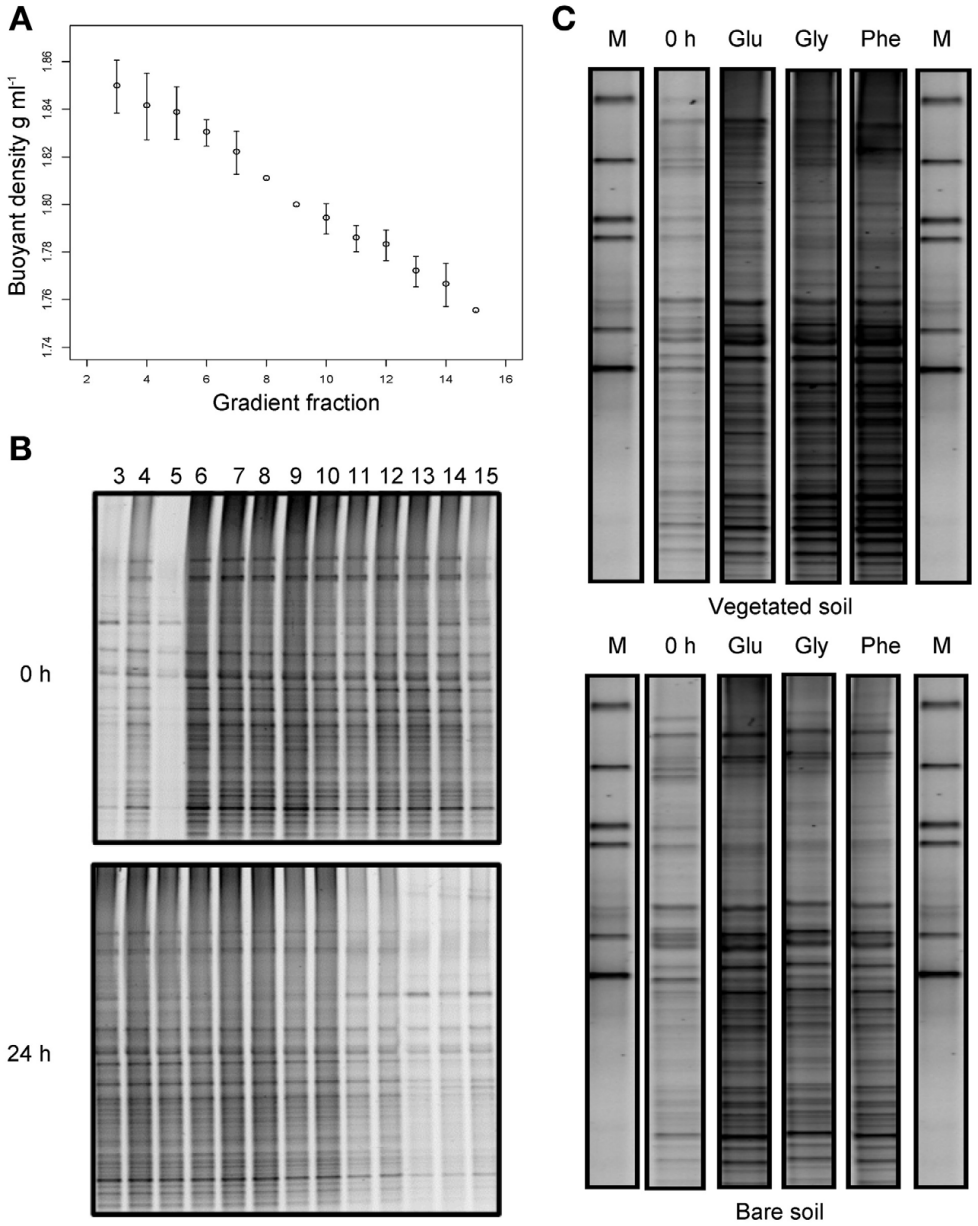
**Stable isotope probing of bacterial communities utilizing ^13^C-labeled substrates.** Buoyant density of extracted RNA in gradient fractions 3–15 following isopycnic density gradient centrifugation **(A)**. Error bars are standard deviations of the mean (*n* = 4). DGGE profiles of bacterial communities present in density gradient fractions 3–15 from vegetated soils at time 0 and 24 h after the addition of ^13^C glucose **(B)**. Density gradient fractions 3–5 were shown to contain ^13^C labeled RNA, as indicated by a general community shift into these fractions after labeled glucose addition. DGGE profiles of bacterial communities present in “heavy” density gradient fraction four from vegetated and bare soils, before and 24 h after the addition of ^13^C-labeled glucose (Glu), glycine (Gly), and phenol (Phe); M denotes marker lanes **(C)**.

To examine the bacterial populations actively utilizing the added substrates, density gradient centrifugation was used to isolate ^13^C enriched RNA, prior to molecular analyses. Consistent with previous studies, reproducible linear gradients were achieved (Figure 5A) spanning a density range of approximately 1.75–1.85 g ml^−1^(Manefield et al., 2002b; Whiteley et al., 2007). We then sought to identify specific fractions containing ^13^C labeled RNA, which could be subsequently examined for all substrate additions. All 20 gradient fractions from unlabeled (0 h) and glucose-labeled (24 h) samples from the vegetated treatment were amplified by reverse transcription PCR. Amplicons were only found in fractions 3–15 (buoyant densities of 1.75–1.85 g ml^−1^) and subsequent DGGE analysis revealed that, despite the presence of some weak bands in the first three “heavy” fractions (3–5) before substrate addition, 24 h after incubation with ^13^C-labeled glucose, DGGE banding patterns were clearly more intense; indicating that ^13^C labeled RNA had been separated into these fractions (Figure 5B). Fraction four (buoyant density 1.84 g ml^−1^ ± 0.013) was then selected to examine the bacterial populations actively utilizing ^13^C-labeled glucose, glycine and phenol prior to (0 h; unlabeled RNA) and 24 h after FOC addition (labeled RNA), through 16S rRNA-SIP and DGGE analyses (Figure 5C). Despite subtle differences in band intensities, the DGGE banding patterns illustrate that most community members originally present in the soil became enriched in substrate ^13^C after 24 h incubation. This suggests that the differences in “total” bacterial communities between vegetated and bare soils are likely indicative of the differences in “active” communities degrading the added FOC, and we found no evidence that limited sub-populations of bacteria were implicated in FOC degradation.

## Discussion

This study combined a ^13^C tracer approach with molecular methodologies to examine FOC and SOC dynamics in relation to vegetation-induced changes in soil resource availability and bacterial communities. Soil C and N contents, microbial biomass and basal respiration were greater in vegetated soil which is consistent with current knowledge on how plants affect soil resource availability and stimulate microbial communities (Nguyen and Guckert, 2001; Orwin et al., 2010; Thomson et al., 2010). Substrate additions revealed that, generally, resources of differing quality were decomposed at different rates, as found in other studies (Webster et al., 1997; Schmidt et al., 2004; Brant et al., 2006; Hartley et al., 2010; Bradford et al., 2013), but rates were consistently higher in vegetated soil independent of substrate type. However, despite bare soils being much less active in degrading added FOC substrates, unlabeled SOC cycling was markedly increased, indicative of positive priming effects. In contrast, unlabeled SOC turnover was retarded in the vegetated soils following the addition of FOC exemplifying negative priming effects. The directions of these priming phenomena were therefore dependent on the presence or absence of plants and associated changes in resource availability and not the type of FOC input used in the assay.

Whilst isotope labeled substrate additions represent a useful assay exploring potential activities with regard to FOC and SOC cycling, such short-term assays can be criticized for not discriminating between “real” or “apparent” priming effects (Blagodatskaya and Kuzyakov, 2008). Additionally, it has been discussed how simple measures of function based on respired C should be used cautiously in inferring longer term dynamics of SOM (Bradford et al., 2008a; Conant et al., 2011). However, we found that the direction of unlabeled SOC turnover following FOC addition reflected longer term changes in soil carbon stocks, where increased SOC cycling in the bare soils was indicative of an overall decrease in soil C content. In the absence of plants microbes must utilize C from an extant pool; and regardless of whether this pool is within existing biomass or native SOC the end result will be C loss (assuming limited inputs from autotrophic carbon fixation). Therefore, based on our results, the short term FOC addition assays proffer a window on these processes, with the directions of priming effects being reflective of longer term changes in soil C. Supporting our findings, increased positive priming effects have been observed in short term assays on forest soils which had reduced soil C content due to the exclusion of plant inputs for 6 years (Brant et al., 2006). More generally, other studies have also reported strongest positive priming effects in soils with comparatively reduced resource availability (Fontaine et al., 2004b; Hamer and Marschner, 2005a,b). Negative priming effects are not documented as frequently as positive ones and whilst there are several potential causes (Kuzyakov et al., 2000; Blagodatskaya et al., 2007) it is thought that they may simply arise through preferential substrate utilization, where organisms switch from degrading SOM to the fresh labile substrate (Sparling et al., 1982; Kuzyakov and Bol, 2006).

Vegetated and bare soils were dominated by *Alphaproteobacteria* and *Acidobacteria*, respectively, in agreement with previous theories that proteobacterial taxa are generally competitively adapted and dominate in soils of higher resource availability, whilst *Acidobacteria* dominate in more resource limited environments (Smit et al., 2001; Griffiths et al., 2003; Cleveland et al., 2007; Fierer et al., 2007; Philippot et al., 2009; Eilers et al., 2010; Thomson et al., 2010; Will et al., 2010). Therefore, we postulate that differences in labile FOC and native SOC processing between vegetated and bare soils may, in addition to simple changes in biomass, be also attributable to changes in dominant taxa. That is, vegetated soils, by virtue of higher resource availability, are dominated by fast growing *r* selected taxa, represented by the *Alphaproteobacteria* which are likely adapted to the fast utilization of labile substrates in the forms of root exudates and rhizodeposits. Conversely, the resource starved bare soils are dominated by *K* selected communities such as the *Acidobacteria*, which are adapted to more efficient, slow growth under limiting conditions. In support of this, a previous experiment (Fierer et al., 2007; Bradford et al., 2008b) directly manipulated forest soil resource availability through repeated sucrose additions, and found low additions generated *Acidobacteria*-dominated communities which were associated with less respiration and long term C loss; whereas high rates of addition favored *Proteobacteria* with increased respiration responses and long term C gain. We feel these findings are analogous to those in our present study, where the removal of plants lowered resource availability, decreased respiratory activity and C storage whilst selecting for an *Acidobacteria*-dominated community. We note that both studies have focused on acidic, low diversity soils which are typically dominated by *Acidobacteria* and *Proteobacteria*, highlighting the need for more distributed studies encompassing different soils and plant communities in order to generalize associations linking plant-driven resource availability, microbial communities and soil carbon storage.

Despite identifying a potentially widespread associative relationship on how resources control bacterial biodiversity and C storage, there remains a challenge to definitively link resource driven changes in communities with explicit roles in C cycling; be it the proposed role of *K* selected taxa in SOC turnover or *r* selected taxa in preferential FOC use. To further investigate the flow of labile FOC into active microbial communities a coupled molecular and isotopic tracer approach was undertaken. Such SIP approaches are believed to shed more light on the explicit mechanisms linking microbes with both FOC and SOC cycling (Bernard et al., 2007, 2012; Pascault et al., 2013). However, we detected few differences between labeled (active) and unlabeled (total) communities 24 h after each substrate amendment indicating many community members had received substrate C, rather than specific active sub-populations. In contrast, a previous study has shown that the addition of labile substrates can shift communities favoring specific bacterial taxa (Goldfarb et al., 2011). However, nearly ten times more C was added in this study compared to ours, potentially explaining why we failed to observe any specific population “enrichment” effects either in our TRFLP or SIP data.

Enrichment of the entire bacterial community has been documented before in soil SIP studies (Rangel-Castro et al., 2005) and is likely due to the universal rapid degradability of simple substrates by soil microbes. Therefore, we found little evidence to suggest that certain discrete populations of bacteria were preferentially utilizing substrates resulting in outgrowth in either vegetated or bare soils. This, therefore, contradicts suppositions that particular members of communities with different life history strategies are exclusively utilizing FOC substrates, but suggests all members are capable of accessing these simple compounds. This is conceivable since, despite an assumed preference for growth in resource limited environments, *K*-strategists are also considered effective at scavenging different substrates (Pianka, 1970; Button, 1993; Bernard et al., 2007; Fierer et al., 2007). Indeed, such theories have been invoked to explain priming effects, whereby easily-assimilated FOC inputs are utilized by *K*-strategists to drive SOC mineralization (Fontaine et al., 2003), though based on the SIP results we were unable to prove or disprove these theories.

The quantitative analyses of ^13^C incorporation into bulk RNA pools revealed further insights into microbial C processing resulting from different substrate additions to vegetated and bare soils. Firstly, we observed that for all soils more glucose was assimilated into RNA than the other two substrates, which is consistent with other studies contrasting glucose, glycine and phenol utilization (Brant et al., 2006; Rinnan and Baath, 2009; Bradford et al., 2013; Frey et al., 2013). Despite these studies using other biomarkers such as fatty acids and total biomass, it is apparent that relatively high glucose-C use efficiency compared to other substrates may be a general phenomenon occurring widely in soils. This may be due to glucose C being preferentially incorporated into structural components (as it requires no extracellular enzymatic breakdown), whereas glycine, as a source of both C and N, gets catabolically metabolized as an energy source (Webster et al., 1997; Hartley et al., 2010) and phenol requires extracellular enzymatic degradation before it can be accessed by the microbial community (Powlowski and Shingler, 1994; Frey et al., 2013). These finding are of wider relevance in demonstrating how different types of FOC inputs are stored or lost from soil and indicate that soil RNA is a potentially useful biomarker for assessing the metabolic fate of different FOC inputs into soil microbial communities.

Secondly, although significant ^13^C incorporation into RNA was found for all samples post-labeling, there were few differences in rates of uptake between vegetated and bare communities. Without a full mass balance calculation these results should be interpreted with some caution; nonetheless it is clear that in vegetated soils a greater proportion of ^13^C was respired, as opposed to assimilated, compared with the bare soils. This can be explained simply by considering past evidence that FOC inputs will be catabolized for energy gain instead of being converted to biomass when C resources are plentiful (Bremer and Kuikman, 1994; Nguyen and Guckert, 2001) or more likely, when N is limiting with respect to plentiful C (Schimel and Weintraub, 2003). Conversely, in bare soil under assumed C limitation (or C limitation with respect to N) these theories suggest more energy from FOC inputs would be channeled into biomass generation. Our findings additionally identify that the biodiversity of soil microbes may also be a factor to consider in explaining these processes, though it is apparent that more quantitative approaches are required to assess the flow of C through specific community members in order to reveal how populations with different life history strategies process distinct C pools. In this regard, further insights into the functional roles played by specific microbial groups in degrading FOC inputs may be gleaned by assessing specific assimilation rates into separated RNA pools using more quantitative approaches such as magnetic bead capture SIP (Macgregor et al., 2002) targeted at specific taxa (e.g., hypothesised *r*- and *K*-strategist taxa). Moreover, to specifically examine SOC degradation and issues pertaining to soil priming, these methods could be coupled with novel experimental approaches to quantitatively trace C from the SOC pool directly (Blagodatskaya et al., 2011). We do, however, also identify an additional complicating issue whereby, particularly for the vegetated soils, substrate “use” may not mean substrate “assimilation” and this should be considered in future soils linking microbial communities and functionality using such isotope tracing approaches.

To conclude, in this upland grassland ecosystem, the field removal of plants for several years decreased soil resources resulting in differential rates of FOC and SOC cycling which are potentially explainable by considering emerging theories on the life histories of microbial taxa. Our results suggest that baseline soil resource availability, controlled by plants, can have a pronounced effect on soil bacterial communities and associated activities in cycling different soil carbon pools. An additional finding of note was that the direction of the priming effect was independent of the type of substrate used, therefore future studies investigating a wider range of soils could perhaps focus on using a single substrate. Coupling isotope and tracer methodologies was useful in providing new insights into the microbial cycling of FOC inputs but further developments are required to quantitatively assess flows into different taxa, and notably trace SOC pools directly. We feel these results provide a strong case for considering microbial biodiversity and the development of further molecular approaches in future studies seeking to understand the differential cycling of different soil carbon pools, be they FOC inputs of different quality or native SOC.

### Conflict of interest statement

The authors declare that the research was conducted in the absence of any commercial or financial relationships that could be construed as a potential conflict of interest.
